# Inferred Mobility-Resolved Resistome Architecture Suggests Recurrent Co-Resistance Modules on a Conserved Chromosomal Backbone in Multidrug-Resistant *Escherichia coli* from Intensive Swine Production in Hungary

**DOI:** 10.3390/antibiotics15040367

**Published:** 2026-04-02

**Authors:** Ádám Kerek, Balázs Nagyházi, Gergely Álmos Tornyos, Levente Hunor Husz, Máté Hetyésy, Eszter Kaszab, Enikő Fehér, Patrik Mag, Ákos Jerzsele

**Affiliations:** 1 Department of Pharmacology and Toxicology, University of Veterinary Medicine, István utca 2, H-1078 Budapest, Hungary; nagyhazi.balazs@student.univet.hu (B.N.); tornyos.gergely.almos@student.univet.hu (G.Á.T.); hetyesy.mate@student.univet.hu (M.H.); mag.patrik@univet.hu (P.M.); jerzsele.akos@univet.hu (Á.J.); 2 National Laboratory of Infectious Animal Diseases, Antimicrobial Resistance, Veterinary Public Health and Food Chain Safety, University of Veterinary Medicine, H-1078 Budapest, Hungaryfeher.eniko@univet.hu (E.F.); 3 One Health Institute, University of Debrecen, Nagyerdei Krt. 98, H-4032 Debrecen, Hungary; 4 Department of Microbiology and Infectious Diseases, University of Veterinary Medicine, István u 2, H-1078 Budapest, Hungary; 5 Department of Bioinformatics, University of Debrecen, Nagyerdei Krt. 98, H-4032 Debrecen, Hungary

**Keywords:** antimicrobial resistance, resistome, mobilome, plasmids, co-selection, swine

## Abstract

**Background:** Multidrug-resistant (MDR) *Escherichia coli* in intensive pig production represents a persistent animal health and One Health concern. Here, we integrated quantitative phenotypic susceptibility data with whole-genome sequencing (WGS) to characterize the resistome and its inferred genomic context (chromosomal vs. plasmid-predicted contigs and mobile genetic element (MGE)-proximal regions) in swine-associated MDR *E. coli* from Hungary. **Methods**: A total of 203 *E. coli* isolates from large-scale pig farms were tested by broth microdilution. Based on resistance-oriented screening from an extended-spectrum β-lactamase (ESBL)-screen-positive pool, 116 isolates were subjected to whole-genome sequencing (WGS) as a resistance-enriched subset. Resistance determinants were annotated using the Comprehensive Antibiotic Resistance Database (CARD). **Results**: Resistance-oriented screening indicated frequent β-lactamase activity and ESBL screening positivity (110/203 and 127/203 isolates, respectively), consistent with strong antimicrobial selection pressure in the source population. Across the full phenotypic panel, 78/203 isolates (38.4%) met the MDR definition (non-susceptible to ≥3 antimicrobial classes), with marked between-farm variation (*p* < 0.001) but no age-group effect (*p* = 0.75). Non-β-lactam minimum inhibitory concentration (MIC) distributions showed pronounced, site-dependent high-MIC “tails”, most notably for tetracyclines, trimethoprim–sulfamethoxazole, fluoroquinolones, and colistin. In the WGS cohort (*n* = 116), we detected 82 distinct resistance determinants (5433 total occurrences), featuring a conserved chromosomal backbone enriched for intrinsic multidrug resistance components and lipid A modification pathways, alongside common plasmid- and MGE-associated acquired ARG modules involving tetracycline (*tetA/tetB*), sulfonamide/trimethoprim (*sul/dfrA*), aminoglycoside-modifying enzymes, and phenicol determinants (*floR/cat*). High-priority mobile determinants were rare but present, including *mcr-1* (3/116; plasmid-associated) and plasmid-mediated quinolone resistance *qnrB5* (2/116). **Conclusions**: Importantly, mobility/context inferences are restricted to this ESBL-screen-enriched WGS subset. Swine-associated *E. coli* from Hungarian large-scale farms harbors complex resistance architectures shaped by co-selection of mobile ARG modules on top of a pervasive chromosomal resistance backbone. Mobility-aware surveillance and stewardship are warranted to mitigate dissemination risks at the animal–environment–human interface.

## 1. Introduction

Antimicrobial resistance (AMR) is among the most urgent global health threats, with resistant infections already causing a substantial mortality burden and projections indicating further escalation in the absence of effective countermeasures [1,2]. The emergence and spread of AMR are tightly coupled to antimicrobial exposure across human, animal, and environmental compartments, where selection can amplify resistant lineages and enrich resistance gene pools even at low or sub-therapeutic concentrations [3,4,5,6]. In food-producing animals, antimicrobial use remains a major driver of selection pressure at the population scale, and global assessments consistently identify intensive livestock systems as hotspots where resistomes can expand and mobilize [4,5,7,8,9].

Swine production is a particularly relevant sector in this context because it frequently relies on group-level interventions and faces recurring bacterial disease challenges across age classes, creating conditions for sustained antimicrobial exposure and repeated selection [10]. In Europe, AMR surveillance and stewardship frameworks explicitly recognize the need to reduce selection pressure in livestock while preserving the effectiveness of medically important antimicrobials [11,12]. The One Health approach provides the conceptual and operational basis for these efforts by emphasizing the inseparability of human, animal, and environmental health and the interconnectivity of AMR reservoirs [12,13,14]. Consistent with this perspective, resistant bacteria and resistance genes can move along multiple routes—direct contact, occupational exposure, manure and slurry dissemination, wastewater and surface water contamination, and ultimately entry into the food chain—forming feedback loops that maintain AMR in circulation even when interventions are implemented in only one compartment [15,16,17].

Within AMR surveillance, *Escherichia coli* occupies a central position because it is both a frequent pathogen and a highly competent genetic “hub” that acquires, maintains, and disseminates antimicrobial resistance genes (ARGs) [11,18,19,20]. Beyond intestinal carriage, *E. coli* includes diverse pathogenic lineages responsible for extraintestinal disease, and its presence across hosts and environments makes it a sensitive indicator for monitoring antimicrobial selection and resistance dissemination [11,21,22,23]. In swine systems, multidrug-resistant (MDR) *E. coli* is not only a therapeutic challenge but also a One Health concern, given evidence that livestock-associated extended-spectrum β-lactamase (ESBL)-producing *E. coli* can be detected in farm environments and in exposed workers, supporting plausible pathways for exchange between animal and human populations [24,25,26,27]. At the European level, ESBL-producing *E. coli* in pigs and pork has been repeatedly documented, underscoring that intensive swine production can act as a reservoir for clinically relevant resistance determinants [24,28,29].

ESBLs are a particularly consequential resistance mechanism because they compromise key β-lactam agents and are often embedded within mobile genetic contexts that facilitate rapid spread [30,31]. The epidemiology of ESBLs is shaped by both clonal expansion and horizontal gene transfer (HGT), with plasmids and other mobile genetic elements (MGEs) enabling the transfer of ESBL determinants across lineages and even across species boundaries [17,24,32,33,34,35,36]. In *E. coli*, MDR phenotypes frequently reflect multilayered architecture: mobile determinants (e.g., ESBL genes and other acquired ARGs) interact with intrinsic or chromosomally encoded mechanisms such as efflux systems and regulatory responses, collectively raising resistance levels and broadening the spectrum of affected drug classes [37,38,39,40]. Moreover, integrons, especially class 1 integrons, can consolidate multiple resistance cassettes and promote co-dissemination of ARG sets under diverse selection pressures [41].

This architecture is directly relevant to co-selection, where exposure to one antimicrobial (or other stressors) can maintain and enrich genetic platforms that also carry resistance to unrelated drug classes [4,17]. In swine-associated *E. coli*, co-selection is often driven by commonly used veterinary drug classes (e.g., tetracyclines, trimethoprim–sulfonamides, and phenicols), which can select for multi-resistance assemblages and stabilize MDR plasmids and MGEs in bacterial populations [42,43,44]. Notably, experimental and field-relevant observations support the notion that phenicol exposure (e.g., florfenicol) may co-select for multiple ARGs in piglets, providing a mechanistic basis for persistence of resistance modules even when certain critically important antimicrobials are used sparingly [44].

Critically important antimicrobials warrant particular attention in *E. coli* because even low-prevalence mobile determinants can have disproportionate clinical and public health impact [11,12]. Fluoroquinolone resistance may emerge through chromosomal target mutations and can also be supported by plasmid-mediated target protection (e.g., *qnr* genes), while multidrug efflux systems contribute to reduced susceptibility and survival under antimicrobial and environmental stresses [37,38,39,40]. Similarly, polymyxins (including colistin) occupy a special position as last-line agents in human medicine and have been tightly scrutinized in animal production due to the risk that mobilizable resistance determinants could disseminate across reservoirs [12]. Plasmid-mediated colistin resistance genes (notably *mcr* variants) have been reported in swine-associated *E. coli* and can co-occur with ESBL determinants, raising concern that mobile platforms may converge multiple high-impact traits [45,46,47]. At the same time, chromosomally encoded lipid A modification pathways can provide a “primed” background that supports persistence and may modulate the resistance phenotype in the presence of additional acquired determinants [46].

Whole-genome sequencing (WGS) has become a cornerstone of high-resolution AMR characterization because it enables simultaneous assessment of strain background, ARG content, and the genetic contexts that shape mobility and dissemination [37,48]. However, a recurring limitation in resistome studies is that ARG cataloguing alone may not resolve where determinants reside (chromosome vs. plasmid vs. mobile genetic element), nor how co-occurrence structures reflect co-occurrence patterns consistent with co-selection and mobilization potential [34,35,49]. Mobility-resolved approaches, combining curated resistome databases with dedicated mobile genetic element (MGE) detection and plasmid-origin inference, are therefore increasingly important to translate genomic findings into actionable risk interpretation for One Health surveillance and antimicrobial stewardship [48,49,50].

Against this background, the aim of the present study was to characterize MDR *E. coli* from large-scale pig farms in Hungary by integrating phenotypic susceptibility profiling with WGS-based, mobility-resolved resistome analysis. Because our broader project addresses β-lactam/ESBL mechanisms in a dedicated companion manuscript, the present work intentionally focuses on MDR patterns and infers genomic context beyond β-lactams. ESBL screening served here as a resistance-enrichment filter for WGS prioritization rather than as the central mechanistic endpoint of this manuscript. We sought to define the acquired resistome across major antimicrobial classes relevant to swine production; assign key resistance determinants to chromosomal, plasmid, or MGE-associated compartments; and identify recurrent co-occurrence patterns consistent with co-selection that may support the persistence and dissemination of MDR in intensive swine production systems.

## 2. Results

### 2.1. Phenotypic Dataset Structure and Multidrug Resistance Burden

A total of 203 *E. coli* isolates were analyzed by broth microdilution. Resistance-oriented screening indicated frequent β-lactamase activity and ESBL screening positivity (110/203 and 127/203 isolates, respectively), providing contextual evidence of strong antimicrobial selection pressure in the source population. Based on this ESBL-oriented pre-screening, isolates were taken forward for short-read WGS as a resistance-enriched subset if they had a complete phenotypic MIC dataset and sufficient genomic DNA quality/quantity for library preparation; 116 isolates ultimately yielded WGS data that could be unambiguously linked to phenotypic records. Accordingly, all resistome and inferred genomic-context analyses reported below apply to this resistance-enriched WGS subset and should not be extrapolated to the broader swine-associated *E. coli* population. ESBL-specific mechanism-level analyses are reported separately, and the present study focuses on MDR patterns beyond β-lactams. Isolates originated from four large-scale production sites (Farm 1, *n* = 58; Farm 2, *n* = 70; Farm 3, *n* = 39; Farm 4, *n* = 36) and represented three age groups (day-old, *n* = 72; 4-week-old, *n* = 65; 6-week-old, *n* = 66). When non-susceptibility was summarized at the antimicrobial-class level, 78/203 isolates (38.4%) met the MDR definition (non-susceptible to ≥3 classes), with a median of 2 resistant classes per isolate (IQR 1–4). MDR prevalence differed significantly between farms (χ^2^ test, *p* = 7.0 × 10^−7^; Benjamini–Hochberg procedure q = 1.2 × 10^−6^), whereas no association was observed across age groups (*p* = 0.75; Benjamini–Hochberg procedure q = 0.84) (Supplementary Table S5). In multivariable logistic regression adjusting for age group, farm remained a strong independent predictor of MDR (Supplementary Table S6, Panel A).

### 2.2. MIC Distributions Reveal Site-Specific Resistance “Tails” Beyond β-Lactams

**Figure 1:**
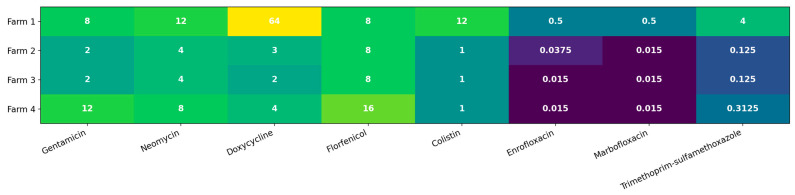
Median minimum inhibitory concentration (MIC) by farm for the non-β-lactam panel. Cell values show the median MIC (µg/mL). Color shading reflects log2-transformed median MIC values (darker colors indicate lower MICs; lighter colors indicate higher MICs).

Across the non-β-lactam panel (Figure 1), MIC distributions were strongly right-shifted for several drug classes, with pronounced site-dependent high-MIC “tails”; full MIC summary is provided in Table 1. For tetracyclines, doxycycline MICs spanned 0.5–128 µg/mL (MIC_50_ = 8 µg/mL; MIC_90_ = 128 µg/mL), and class-level non-susceptibility was frequent (95/203; 46.8%). Aminoglycoside resistance occurred at moderate levels (54/203; 26.6%), with gentamicin MIC_50/90_ of 2/32 µg/mL and neomycin MIC_50/90_ of 4/64 µg/mL (Table 1). Folate-pathway inhibitor non-susceptibility (trimethoprim–sulfamethoxazole) affected 66/203 isolates (32.5%), showing a distinctly bimodal distribution and a high-MIC tail (trimethoprim–sulfamethoxazole MIC_90_ = 256 µg/mL).

Two drug classes displayed particularly striking tails. First, polymyxin (colistin) MICs ranged from 0.5 to 32 µg/mL (MIC_50_ = 1 µg/mL; MIC_90_ = 32 µg/mL), and elevated MICs clustered strongly by site: Farm 1 accounted for the majority of isolates with colistin MIC ≥ 16 µg/mL. Second, fluoroquinolone distributions showed discrete high-level subpopulations: 14 isolates exhibited enrofloxacin MIC = 32 µg/mL and 12 isolates had marbofloxacin MIC = 32 µg/mL, again concentrated predominantly in Farm 1 (Figure 1). Together, these patterns indicate that non-β-lactam resistance is not merely a background feature of the collection, but is structured by production site, consistent with localized selective regimes and/or dissemination dynamics.

High-MIC tail endpoints were strongly farm-associated for colistin (colistin ≥ 16 µg/mL; *p* = 1.0 × 10^−14^; q = 6.1 × 10^−14^) and for fluoroquinolones (enrofloxacin ≥ 32 µg/mL: *p* = 3.4 × 10^−8^; q = 8.3 × 10^−8^; marbofloxacin ≥ 32 µg/mL: *p* = 5.5 × 10^−7^; q = 1.1 × 10^−6^) (Supplementary Table S5). Among additional endpoints, trimethoprim–sulfamethoxazole high-level non-susceptibility (≥256 µg/mL) and high doxycycline MICs (≥128 µg/mL) also differed by farm (q ≤ 0.0034), whereas age-group associations were limited (Supplementary Table S5). Effect-size modelling confirmed that Farm 1 status remained strongly associated with the colistin high-MIC tail, and that farms differed in the odds of the high doxycycline and TMP–SMX tail endpoints after adjustment for age (Supplementary Table S6, Panel A).

### 2.3. Genomic Resistome Size Is Dominated by a Conserved Chromosomal Backbone

**Figure 2:**
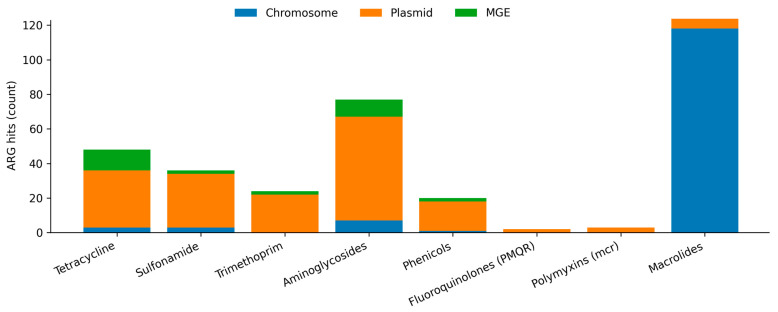
Distribution of Comprehensive Antibiotic Resistance Database (CARD)-annotated resistance determinant hits across antimicrobial classes and genomic compartments (chromosome, plasmid, and MGE-associated), with mobile genetic elements (MGE) association assigned based on MobileElementFinder-defined proximity to predicted mobile elements. ARG—antimicrobial resistance gene.

Whole-genome resistome and mobility analyses were performed for 116 isolates that could be unambiguously linked to phenotyped isolates. Across these genomes, 82 distinct Comprehensive Antibiotic Resistance Database (CARD)-annotated resistance determinants were detected, comprising 5433 total hits. The per-isolate resistome was highly conserved in size (median 46 unique determinants; IQR: 45–47), reflecting a strong shared chromosomal backbone rather than extensive acquisition-driven diversification (Figure 2). Importantly, this conserved determinant count largely reflects ubiquitous chromosomal entries in CARD (e.g., intrinsic efflux/regulatory and envelope-associated components) and therefore should not be interpreted as a proxy for phenotypic MDR severity. Instead, phenotypic variability is more plausibly driven by differences in the presence and composition of acquired accessory ARG modules and their inferred genomic context and chromosomal variation and regulatory/permeability states that can shift MICs without changing acquired-ARG presence/absence. Consistent with this, 93.0% of hits were chromosomal (5055/5433), while only 5.9% (322/5433) mapped to plasmid-predicted contigs, and 1.0% (56/5433) were classified as mobile-element-associated based on proximity to MGEs. A class-level summary of non-β-lactam resistance determinants stratified by genomic context (chromosome/MGE/plasmid) is provided in Supplementary Table S1.

Chromosomal hits were enriched for multidrug efflux and regulatory components as well as lipid A modification pathways. Notably, determinants implicated in polymyxin response and envelope remodeling (e.g., *eptA*, *pmrF*) were ubiquitous, supporting the interpretation that colistin phenotype cannot be read out from gene presence/absence alone in this background, but is likely influenced by chromosomal mechanisms not captured by acquired-ARG presence/absence alone (e.g., regulatory variation and/or target-site alterations), which were not directly resolved in this study.

### 2.4. Inferred Genomic Context of Acquired ARGs Reveals Recurrent Co-Occurrence Patterns Consistent with Co-Selection

**Figure 3:**
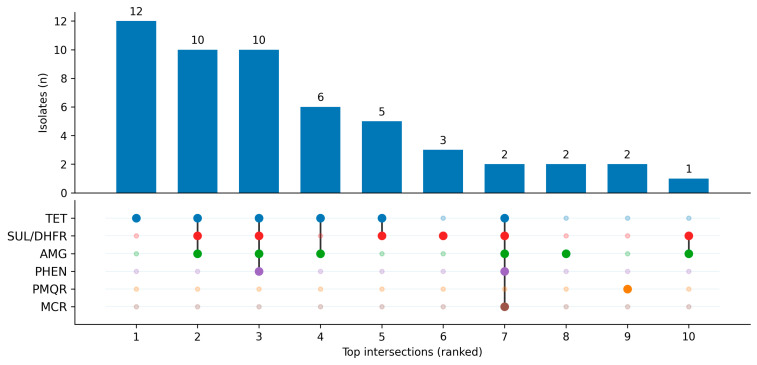
UpSet-style summary of co-occurrence patterns among key non-β-lactam resistance gene groups in the whole-genome sequencing (WGS) cohort. Bars show the number of isolates per intersection (top-ranked combinations). In the matrix, large filled circles indicate presence and small faint circles indicate absence of a gene group; vertical lines connect co-occurring groups within each intersection. Matrix colors are row-specific and used to distinguish gene groups (no additional biological variable is encoded). Gene groups: TET (*tet*), SUL/DHFR (*sul/dfr*), AMG (aminoglycoside genes), PHEN (phenicol genes), PMQR (plasmid-mediated quinolone resistance genes), MCR (*mcr-1*).

Despite the dominance of chromosomal determinants, acquired non-β-lactam ARGs were common at the isolate level, and 90/116 genomes carried at least one mobile-associated determinant from tetracycline, folate-pathway, aminoglycoside, or phenicol categories. Key acquired non-β-lactam resistance determinants and their compartment assignments are summarized in Supplementary Table S2. These genes frequently appeared in recurrent co-occurrence patterns that are consistent with co-selection, although physical linkage and precise genomic location cannot be directly demonstrated with short-read data. The most frequent module linked sulfonamide/trimethoprim resistance (*sul1/sul2* with *dfrA* variants) with aminoglycoside-modifying enzymes (e.g., *aadA2*, *ANT(3″)-IIa*), consistent with class 1 integron-like assemblages; for example, *dfrA1* co-occurred with *sul1* in 13 genomes and *sul2* co-occurred with *ANT(3″)-IIa* in 11 genomes. Tetracycline resistance determinants were also recurrent (*tetA* in 29/116; *tetB* in 12/116) and frequently appeared alongside aminoglycoside genes, reinforcing a co-selection architecture rather than single-drug specialization (Figure 3).

**Figure 4:**
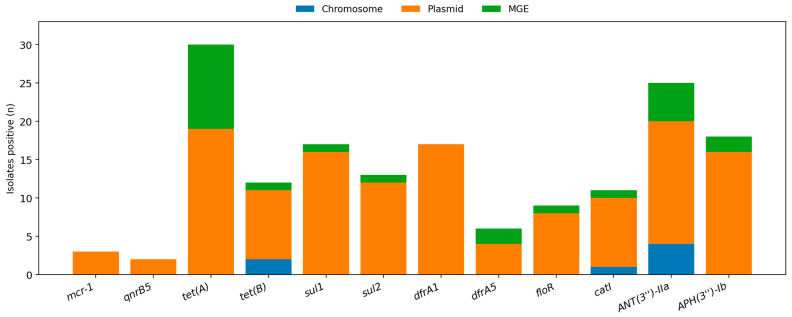
Critical determinants panel showing isolates positive (stacked by genomic context) for priority acquired genes, including *mcr-1*, *qnrB5*, and co-resistance determinants (*tet*, *sul/dfr*, aminoglycosides, phenicols).

Critically, One Health-relevant “sentinel” mobile determinants were rare in this MDR background; plasmid-mediated colistin resistance (*mcr-1*) was detected in only 3/116 genomes and plasmid-mediated quinolone resistance (*qnrB5*) in 2/116 genomes. This scarcity, juxtaposed with the phenotypic tails observed for colistin and fluoroquinolones, points to a predominantly chromosomal (and likely mutational/regulatory) basis for high-MIC subpopulations in these classes within the broader collection. MGE-proximal acquired ARGs identified by MobileElementFinder are listed in Supplementary Table S3. The prevalence and genomic localization of selected high-priority determinants are summarized in Figure 4.

### 2.5. Phenotype–Genotype Agreement Is Class-Dependent and Limited by Chromosomal Mechanisms

Mobility-resolved genotyping showed strong class-dependent predictive performance when benchmarked against phenotypes in the WGS-linked subset. For tetracyclines, presence of *tet* genes captured a substantial fraction of phenotypic resistance (sensitivity: 69.6%; specificity: 91.7%; accuracy: 81.0%). For folate-pathway inhibitors, requiring concurrent *sul* and *dfrA* improved specificity (97.7%) and yielded high overall accuracy (84.5%), consistent with the observed co-selection of these determinants in recurrent modules. In contrast, aminoglycosides showed weaker agreement (accuracy: 71.6%), reflecting heterogeneous gene content and the potential contribution of dosage effects, gene expression context, and/or breakpoint-adjacent MIC distributions (Supplementary Table S4).

For polymyxins, *mcr-1* was perfectly specific but poorly sensitive (sensitivity: 42.9%; specificity: 100%), explaining only a minority of phenotypically non-susceptible isolates; the remaining cases lacked plasmid-mediated markers, suggesting that elevated colistin MICs are not predominantly mcr-driven in this collection. Similarly, the rarity of *qnrB5* relative to the observed fluoroquinolone MIC tail suggests that acquisition is not the primary driver of fluoroquinolone non-susceptibility in this dataset. Because mutation-level analyses of canonical chromosomal loci (e.g., *pmrAB*, *phoPQ*, *mgrB*, *gyrA*, and *parC*) were outside the scope of this work, the contribution of chromosomal mechanisms is inferred rather than directly confirmed.

## 3. Discussion

This study provides an integrated phenotypic–genomic snapshot of antimicrobial resistance in *E. coli* isolated from large-scale pig production in Hungary, with a deliberate emphasis on the broader MDR burden and the genomic architecture that may sustain co-resistance across antimicrobial classes. By combining standardized MIC data with WGS-based resistome profiling and an inference framework for genomic context (chromosome vs. plasmid-predicted contigs vs. MGE proximity), we interpret resistance in this setting as a layered system: acquired determinants assigned to plasmid-predicted or MGE-proximal contexts represent a potentially transferable component, whereas a pervasive chromosomal background of intrinsic and regulatory mechanisms may modulate expression, persistence, and phenotypic outcomes. This framing aligns with One Health concepts linking antimicrobial exposure and AMR evolution across animal, human, and environmental compartments [13].

The phenotypic burden and between-farm heterogeneity observed here are consistent with sustained selection pressure in intensive pig production, where usage patterns can be intense and historically entrenched [7,10,11]. Under such conditions, resistant commensal *E. coli* can persist as herd-associated reservoirs and contribute to onward dissemination via manure and the farm environment, with implications at the animal–environment–human interface [19]. Notably, MDR prevalence did not differ by age group, which is compatible with early-life acquisition and persistence of resistant lineages and with farm-level drivers (management, antimicrobial exposure, local transmission) dominating cross-sectional age effects when MDR is defined at the antimicrobial-class level.

The prominence of sul determinants parallels reports from pig-associated *E. coli* and pig production environments, where *sul* genes are commonly plasmid-associated and often embedded in integron-like structures [42,51,52]. Likewise, the widespread occurrence of tetracycline determinants is consistent with their repeatedly high prevalence across animal and human *E. coli* backgrounds and with long-standing tetracycline exposure as an ecological driver [53]. The phenicol axis is compatible with evidence that florfenicol exposure can co-select broader resistance repertoires and sustain MDR even when pathogen pressure fluctuates, consistent with molecular descriptions of florfenicol-resistant pig-associated *E. coli* [43,44]. Together, these patterns support a co-selection interpretation at the level of co-occurrence and inferred context, while avoiding overstatement about demonstrable linkage [4].

High-priority determinants were rare but epidemiologically meaningful. PMQR (*qnr*) was detected at low frequency, consistent with variable baseline levels across production settings and sampling frames [54]. Importantly, our data also highlight that fluoroquinolone phenotypes cannot be interpreted solely from PMQR presence/absence, because chromosomal efflux and regulatory systems can shift susceptibility and contribute to genotype–phenotype discordance [37,38,40]. A similar duality was observed for polymyxins: plasmid-borne *mcr-1* occurred only in a small subset, consistent with reports that *mcr* can be infrequent yet of high One Health relevance due to mobility and international spread [46,55]. In parallel, chromosomal lipid A modification capacity is widespread in Enterobacterales, and the combination of low *mcr* prevalence with a phenotypic high-MIC tail is compatible with a substantial chromosomal contribution that warrants targeted follow-up (e.g., mutation screening and/or expression-aware approaches) [56]. Even low-prevalence *mcr*-positive isolates remain surveillance priorities because mobile platforms can disseminate across interfaces and may be maintained by co-selection [32,34].

From a translational perspective, an important implication is that WGS-based resistance prediction is inherently class-dependent: it is typically robust for well-annotated acquired determinants, but less deterministic when chromosomal regulation, efflux, permeability, and expression state become decisive. Benchmarking work indicates that systematic discordances can arise from incomplete databases, assembly artifacts, variable expression, and breakpoint-adjacent phenotypes [57], and efflux is a canonical case where gene presence does not translate into a simple binary rule [37,38,40]. Therefore, the most defensible surveillance strategy remains integrated: quantitative MIC phenotyping provides the anchor, while WGS adds mechanism space, inferred mobility context, and co-selection risk [48,57].

Several limitations are important for inference. First, the WGS cohort represents a resistance-enriched subset derived from an ESBL-screen-positive pool; accordingly, resistome composition and inferred genomic context should not be generalized beyond this selected subset. Second, short-read WGS constrains plasmid resolution and can blur precise localization in mosaic regions; therefore, genomic context and “mobility” should be interpreted as inferred, ideally complemented by long-read sequencing in follow-up work [34,49]. Third, we did not conduct mutation-level screening of canonical chromosomal loci associated with polymyxin and fluoroquinolone resistance; thus, chromosomal contributions are inferred from MIC distributions and gene-content patterns rather than directly confirmed. Despite these constraints, the combined phenotype–genotype view supports an ecological interpretation in which MDR persistence reflects co-occurring acquired determinants superimposed on a pervasive chromosomal background that can modulate expression and tolerance [4,37,38].

Taken together, our findings place the sampled large-scale pig farms within a broader European and global landscape in which MDR *E. coli* is sustained by the interplay of acquired resistance determinants and chromosomal adaptive capacity [7,10,11,19,58], underscoring the One Health value of surveillance that integrates phenotype, WGS, and inferred context while explicitly accounting for co-selection dynamics [4,34,57].

## 4. Materials and Methods

### 4.1. Study Setting and Bacterial Isolates

Archived *Escherichia coli* isolates were retrieved in late 2023 from an existing diagnostic strain collection comprising isolates originating from four large-scale pig farms in Hungary (Farm 1, Farm 2, Farm 3, Farm 4). The original clinical specimens had been collected by the attending farm veterinarians as part of routine diagnostic procedures and submitted to the Department of Epidemiology and Microbiology, University of Veterinary Medicine Budapest, for bacteriological examination. Following routine isolation and identification, pure *E. coli* cultures were stored at −80 °C using the Microbank bead preservation system (Pro-Lab Diagnostics, Richmond Hill, ON, Canada) and were provided for the present study.

### 4.2. Minimum Inhibitory Concentration (MIC) Determination

Prior to selecting isolates for whole-genome sequencing (WGS), MIC testing was performed according to the Clinical and Laboratory Standards Institute (CLSI) methodology guidelines, and resistance breakpoints were interpreted using CLSI criteria [59]. Frozen isolates were resuspended the day before testing in 3 mL cation-adjusted Mueller–Hinton broth (CAMHB) and incubated at 37 °C for 18–24 h. Broth microdilution was performed in 96-well microtiter plates (VWR International, LLC., Debrecen, Hungary) containing 90 µL CAMHB per well.

Antibiotic stock solutions (Merck KGaA, Darmstadt, Germany) were prepared at 1024 µg/mL in accordance with CLSI recommendations, and two-fold serial dilutions were generated across the plate [59]. After preparation of the dilution series, excess liquid was removed after column 10 to maintain a final volume of 90 µL per well.

Bacterial suspensions were adjusted to 0.5 McFarland using a nephelometer (Thermo Fisher Scientific, Budapest, Hungary), and 10 µL inoculum was added to each well as described in the applied protocol. MICs were read using the Sensititre SWIN automated reading system and the VIZION system (v3.4; Thermo Fisher Scientific, Budapest, Hungary). Quality control was ensured using the reference strain *E. coli* ATCC 25922.

### 4.3. Phenotypic ESBL Confirmation

Phenotypic detection of extended-spectrum β-lactamase (ESBL) production was performed according to a CLSI-recommended approach [60]. MICs were determined for cefotaxime and for the cefotaxime–clavulanic acid combination, with a constant clavulanic acid concentration of 4 µg/mL across all dilution steps. Plates were incubated at 37 °C for 18–24 h and interpreted according to the CLSI definition: an isolate was considered ESBL-producing when the MIC measured for the antibiotic–clavulanic acid combination showed a ≥3 two-fold dilution decrease (i.e., ≥8-fold) compared with the antibiotic alone (e.g., cefotaxime MIC 32 µg/mL vs. cefotaxime–clavulanic acid MIC 2 µg/mL).

### 4.4. DNA Extraction, Library Preparation, and Whole-Genome Sequencing

The WGS cohort represents a resistance-enriched subset derived from the ESBL-screen-positive pool. Isolates were taken forward for short-read WGS if they had a complete phenotypic MIC dataset and sufficient genomic DNA quantity/quality for library preparation; 116 isolates produced usable sequencing data that could be unambiguously linked to corresponding phenotypic records. However, all downstream analyses in this manuscript were designed to characterize MDR patterns and mobility-resolved resistome architecture beyond β-lactams. Genomic DNA was extracted using the Zymo Quick-DNA Fungal/Bacterial Miniprep Kit (Zymo Research, Irvine, CA, USA) following the manufacturer’s instructions [61].

Mechanical lysis was performed by bead beating using a Qiagen TissueLyzer LT (Qiagen GmbH, Hilden, Germany) at 50 Hz for 5 min. Extracted DNA was stored at −20 °C until sequencing. Paired-end sequencing was performed by Novogene on an Illumina NextSeq 500 platform. Illumina sequencing was conducted using a bridge amplification-based paired-end approach. Libraries were prepared using the Illumina Nextera XT DNA Library Preparation Kit [62] and indexed with the Nextera XT Index Kit v2 Set A (i5 and i7 primer pairs). Input DNA was diluted to a final concentration of 0.2 ng/µL (2.5 µL), followed by the addition of Tagment DNA buffer (5 µL) and Amplicon Tagment Mix (2.5 µL).

Tagmentation was performed at 55 °C for 6 min in an Eppendorf Mastercycler nexus GX2 (Eppendorf SE, Hamburg, Germany), followed by cooling to 10 °C.

### 4.5. Read Processing and Genome Assembly

Raw sequencing data quality was evaluated using FastQC (v0.11.9) [63]. Read preprocessing was performed with fastp (v0.23.2-3), including quality filtering and adapter trimming as applicable [64]. Error correction was conducted using Bloocoo (v1.0.7) [65]. Additional trimming steps were performed with Trim Galore (v0.6.6) [66].

De novo assembly was performed using MEGAHIT (v1.2.9) [67]. Read merging for assembly optimization was performed using GAM-NGS (v1.1b) [68].

Assembled contigs were validated using QUAST (v5.2) and BUSCO (v5) to assess assembly quality and completeness [69,70]. Basic genome properties were estimated using GenomeScope (v2.2) based on k-mer distributions (e.g., genome size and coverage characteristics) [71]. Species confirmation and quality checks of the assemblies were performed using CheckM (v1.2.2) [72] and Kraken (v1.1.1) [73].

Open reading frames (ORFs) were predicted using Prodigal (v2.6.3) [74]. Antimicrobial resistance genes (ARGs) were identified using Resistance Gene Identifier (RGI; v5.1.0) and ABRicate based on the CARD [48]. Only hits meeting the CARD “STRICT” criteria and showing ≥90% sequence identity and ≥90% coverage were retained for downstream analyses.

Association of detected ARGs with mobile genetic elements (MGEs) was assessed using MobileElementFinder (v1.0.3) [49]. An ARG was classified as MGE-associated if it was located within the maximum transposon distance defined in the MobileElementFinder database for the corresponding microorganism. Plasmid origin of contigs was inferred using PlasFlow (v1.1) [50].

For both MGE- and plasmid-related outputs, only predictions supported by ≥10,000 bp identified sequence were considered. To enable compartmentalized reporting (chromosome vs. plasmid vs. MGE), ARGs were assigned using a single-compartment rule for figures/tables: MGE-associated if MobileElementFinder-positive, otherwise plasmid-associated if located on a contig classified as plasmid by PlasFlow (≥10 kb criterion), otherwise chromosomal.

### 4.6. Statistical Analysis

Associations between categorical outcomes (e.g., MDR status; class-level non-susceptibility; predefined high-MIC tail endpoints) and farm or age group were tested using χ^2^ tests or Fisher’s exact tests, as appropriate based on expected cell counts. Where multiple related comparisons were performed, *p*-values were adjusted using the Benjamini–Hochberg procedure. For selected endpoints, multivariable logistic regression models were fitted to estimate effect sizes (odds ratios with 95% confidence intervals) for farm and age group; these results are reported in Supplementary Table S6. All tests were two-sided, with *p* < 0.05 considered statistically significant.

## 5. Conclusions

Overall, this study documents a substantial MDR burden among *E. coli* isolates from four large-scale pig farms in Hungary, with pronounced between-farm heterogeneity and class-specific high-MIC subpopulations for several non-β-lactam agents. In a resistance-enriched WGS subset derived from an ESBL-screen-positive pool (*n* = 116), we observed a conserved chromosomal backbone of CARD-annotated determinants alongside recurrent acquired ARG constellations that were frequently assigned to plasmid-predicted contigs or MGE-proximal regions using an inference-based context framework.

These findings support the practical value of integrating quantitative MIC phenotyping with WGS-based resistome profiling while underscoring important limits of inference from short-read assemblies: genomic context and mobility should be interpreted as inferred rather than directly demonstrated, and genotype–phenotype prediction remains class-dependent, particularly where chromosomal mechanisms dominate. Follow-up work using long-read sequencing and targeted mutation/expression analyses will be important to resolve linkage and to refine phenotype–genotype mapping for surveillance and stewardship in intensive swine production systems.

## Figures and Tables

**Table 1:** Minimum inhibitory concentration (MIC) summary statistics (full panel).

Antibiotics	*n*	MIC_min_	MIC_50_	MIC_90_	MIC_max_
Amoxicillin	203	1	128	128	128
Amoxicillin–clavulanic acid	203	1	4	16	32
Cefotaxime	203	0.06	2	128	128
Cefotaxime–clavulanic acid	203	0.06	0.06	128	128
Ceftiofur	203	0.06	4	32	32
Cefquinome	203	0.015	2	32	32
Gentamicin	203	0.5	2	32	128
Neomycin	203	1	4	64	128
Doxycycline	203	0.5	8	128	128
Florfenicol	203	2	8	32	128
Colistin	203	0.5	1	32	32
Enrofloxacin	203	0.015	0.06	2	32
Marbofloxacin	203	0.015	0.015	2	32
Trimethoprim–sulfamethoxazole	203	0.125	0.125	256	256

## Data Availability

The whole-genome sequencing data generated in this study have been deposited in the NCBI BioProject repository under accession PRJNA1414074. The remaining data are available from the corresponding author upon reasonable request.

## Supplementary Materials

The following supporting information can be downloaded at: https://www.mdpi.com/article/10.3390/antibiotics15040367/s1. Table S1: Genomic context of non-β-lactam resistance determinants by antimicrobial class in the whole-genome sequencing (WGS) cohort. Table S2: Key acquired antimicrobial resistance determinants and genomic context in the whole-genome sequencing (WGS) cohort. Table S3: Mobile genetic element (MGE)-associated acquired antimicrobial resistance genes (ARGs). Table S4: Phenotype–genotype agreement metrics for whole-genome sequencing (WGS)-based resistance prediction in the WGS-linked subset. Table S5: Farm- and age-group associations for multidrug-resistant (MDR) and selected phenotypic endpoints. Table S6: Effect-size analyses for farm- and age-associated phenotypic endpoints (multivariable logistic regression) and farm-level prevalence/associations of key acquired antimicrobial resistance gene (ARG) groups in the whole-genome sequencing (WGS) subset.

## References

[1] Van Oort C.M., Ferrell J.B., Remington J.M., Wshah S., Li J. (2021). AMPGAN v2: Machine Learning Guided Design of Antimicrobial Peptides. J. Chem. Inf. Model..

[2] Agyare C., Boamah V.E., Zumbi C.N., Osei F.B., Kumar Y. (2018). Antibiotic Use in Poultry Production and Its Effects on Bacterial Resistance. Antimicrobial Resistance—A Global Threat.

[3] Patel S.J., Wellington M., Shah R.M., Ferreira M.J. (2020). Antibiotic Stewardship in Food-Producing Animals: Challenges, Progress, and Opportunities. Clin. Ther..

[4] You Y., Silbergeld E.K. (2014). Learning from Agriculture: Understanding Low-Dose Antimicrobials as Drivers of Resistome Expansion. Front. Microbiol..

[5] Thanner S., Drissner D., Walsh F. (2016). Antimicrobial Resistance in Agriculture. mBio.

[6] Benmazouz I., Kövér L., Kardos G. (2024). The Rise of Antimicrobial Resistance in Wild Birds: Potential AMR Sources and Wild Birds as AMR Reservoirs and Disseminators: Literature Review. Magy. Állatorvosok Lapja.

[7] Van Boeckel T.P., Brower C., Gilbert M., Grenfell B.T., Levin S.A., Robinson T.P., Teillant A., Laxminarayan R. (2015). Global Trends in Antimicrobial Use in Food Animals. Proc. Natl. Acad. Sci. USA.

[8] Lőrincz E.É., Wagenhoffer Z., Zenke P. (2025). The relevance of epigenetic research in veterinary sciences: Literature review. Magy. Állatorvosok Lapja.

[9] Varga-Balogh O., Olasz F. (2025). Role of microbiota in cattle reproduction: Literature review. Magy. Állatorvosok Lapja.

[10] Lekagul A., Tangcharoensathien V., Yeung S. (2019). Patterns of Antibiotic Use in Global Pig Production: A Systematic Review. Vet. Anim. Sci..

[11] Aerts M., Battisti A., Hendriksen R., Kempf I., Teale C., Tenhagen B.-A., Veldman K., Wasyl D., Guerra B., Liébana E. (2019). Technical Specifications on Harmonised Monitoring of Antimicrobial Resistance in Zoonotic and Indicator Bacteria from Food-Producing Animals and Food. EFSA J..

[12] Aidara-Kane A., Angulo F.J., Conly J.M., Minato Y., Silbergeld E.K., McEwen S.A., Collignon P.J., WHO Guideline Development Group (2018). World Health Organization (WHO) Guidelines on Use of Medically Important Antimicrobials in Food-Producing Animals. Antimicrob. Resist. Infect. Control.

[13] Danasekaran R. (2024). One Health: A Holistic Approach to Tackling Global Health Issues. Indian J. Community Med..

[14] Farkas Z., Csorba S., Vribék K., Süth M., Czudor Z., Tényi Á., Jóźwiak Á. (2025). Establishing a data infrastructure system for veterinary public health and food chain safety data through the development of a repositioning platform. Magy. Állatorvosok Lapja.

[15] Kim J., Ahn J. (2022). Emergence and Spread of Antibiotic-Resistant Foodborne Pathogens from Farm to Table. Food Sci. Biotechnol..

[16] Ma L., Li B., Jiang X.-T., Wang Y.-L., Xia Y., Li A.-D., Zhang T. (2017). Catalogue of Antibiotic Resistome and Host-Tracking in Drinking Water Deciphered by a Large Scale Survey. Microbiome.

[17] Founou L.L., Founou R.C., Essack S.Y. (2016). Antibiotic Resistance in the Food Chain: A Developing Country-Perspective. Front. Microbiol..

[18] Silva A., Silva V., Pereira J.E., Maltez L., Igrejas G., Valentão P., Falco V., Poeta P. (2023). Antimicrobial Resistance and Clonal Lineages of *Escherichia coli* from Food-Producing Animals. Antibiotics.

[19] Hammerum A.M., Heuer O.E. (2009). Human Health Hazards from Antimicrobial-Resistant *Escherichia coli* of Animal Origin. Clin. Infect. Dis..

[20] Farkas M., Könyves L., Csorba S., Farkas Z., Józwiák Á., Süth M., Kovács L. (2024). Biosecurity Situation of Large-Scale Poultry Farms in Hungary According to the Databases of National Food Chain Safety Office Centre for Disease Control and Biosecurity Audit System of Poultry Product Board of Hungary in the Period of 2021–2022. Magy. Állatorvosok Lapja.

[21] Kaper J.B., Nataro J.P., Mobley H.L.T. (2004). Pathogenic *Escherichia coli*. Nat. Rev. Microbiol..

[22] Martinez-Medina M. (2021). Special Issue: Pathogenic *Escherichia coli*: Infections and Therapies. Antibiotics.

[23] Russo T.A., Johnson J.R. (2000). Proposal for a New Inclusive Designation for Extraintestinal Pathogenic Isolates of *Escherichia coli*: ExPEC. J. Infect. Dis..

[24] Ewers C., Bethe A., Semmler T., Guenther S., Wieler L.H. (2012). Extended-Spectrum β-Lactamase-Producing and AmpC-Producing *Escherichia coli* from Livestock and Companion Animals, and Their Putative Impact on Public Health: A Global Perspective. Clin. Microbiol. Infect..

[25] Dahms C., Hübner N.-O., Kossow A., Mellmann A., Dittmann K., Kramer A. (2015). Occurrence of ESBL-Producing *Escherichia coli* in Livestock and Farm Workers in Mecklenburg-Western Pomerania, Germany. PLoS ONE.

[26] Dohmen W., Bonten M.J.M., Bos M.E.H., van Marm S., Scharringa J., Wagenaar J.A., Heederik D.J.J. (2015). Carriage of Extended-Spectrum β-Lactamases in Pig Farmers Is Associated with Occurrence in Pigs. Clin. Microbiol. Infect..

[27] Somogyi F., Farkas O. (2025). Possibilities of in vitro Modelling of Porcine Infectious Enteropathies: Literature review. Magy. Állatorvosok Lapja.

[28] Meissner K., Sauter-Louis C., Heiden S.E., Schaufler K., Tomaso H., Conraths F.J., Homeier-Bachmann T. (2022). Extended-Spectrum ß-Lactamase-Producing *Escherichia coli* in Conventional and Organic Pig Fattening Farms. Microorganisms.

[29] Bergšpica I., Kaprou G., Alexa E.A., Prieto M., Alvarez-Ordóñez A. (2020). Extended Spectrum β-Lactamase (ESBL) Producing *Escherichia coli* in Pigs and Pork Meat in the European Union. Antibiotics.

[30] Paterson D.L., Bonomo R.A. (2005). Extended-Spectrum β-Lactamases: A Clinical Update. Clin. Microbiol. Rev..

[31] Bradford P.A. (2001). Extended-Spectrum β-Lactamases in the 21st Century: Characterization, Epidemiology, and Detection of This Important Resistance Threat. Clin. Microbiol. Rev..

[32] Levy S.B., Fitzgerald G.B., Macone A.B. (1976). Spread of Antibiotic-Resistant Plasmids from Chicken to Chicken and from Chicken to Man. Nature.

[33] Leclerc Q.J., Lindsay J.A., Knight G.M. (2019). Mathematical Modelling to Study the Horizontal Transfer of Antimicrobial Resistance Genes in Bacteria: Current State of the Field and Recommendations. J. R. Soc. Interface.

[34] Vrancianu C.O., Popa L.I., Bleotu C., Chifiriuc M.C. (2020). Targeting Plasmids to Limit Acquisition and Transmission of Antimicrobial Resistance. Front. Microbiol..

[35] Shintani M., Sanchez Z.K., Kimbara K. (2015). Genomics of Microbial Plasmids: Classification and Identification Based on Replication and Transfer Systems and Host Taxonomy. Front. Microbiol..

[36] Bush K., Bradford P.A. (2020). Epidemiology of β-Lactamase-Producing Pathogens. Clin. Microbiol. Rev..

[37] Kumar A., Schweizer H.P. (2005). Bacterial Resistance to Antibiotics: Active Efflux and Reduced Uptake. Adv. Drug Deliv. Rev..

[38] Li X.-Z., Nikaido H. (2009). Efflux-Mediated Drug Resistance in Bacteria: An Update. Drugs.

[39] Piddock L.J.V. (2006). Clinically Relevant Chromosomally Encoded Multidrug Resistance Efflux Pumps in Bacteria. Clin. Microbiol. Rev..

[40] Li X.-Z., Plésiat P., Nikaido H. (2015). The Challenge of Efflux-Mediated Antibiotic Resistance in Gram-Negative Bacteria. Clin. Microbiol. Rev..

[41] Karimi Dehkordi M., Halaji M., Nouri S. (2020). Prevalence of Class 1 Integron in *Escherichia coli* Isolated from Animal Sources in Iran: A Systematic Review and Meta-Analysis. Trop. Med. Health.

[42] Mazurek J., Bok E., Stosik M., Baldy-Chudzik K. (2015). Antimicrobial Resistance in Commensal *Escherichia coli* from Pigs during Metaphylactic Trimethoprim and Sulfamethoxazole Treatment and in the Post-Exposure Period. Int. J. Environ. Res. Public Health.

[43] Blickwede M., Schwarz S. (2004). Molecular Analysis of Florfenicol-Resistant *Escherichia coli* Isolates from Pigs. J. Antimicrob. Chemother..

[44] Holman D.B., Gzyl K.E., Kommadath A. (2024). Florfenicol Administration in Piglets Co-Selects for Multiple Antimicrobial Resistance Genes. mSystems.

[45] Pungpian C., Angkititrakul S., Chuanchuen R. (2022). Genomic Characterization of Antimicrobial Resistance in mcr-Carrying ESBL-Producing *Escherichia coli* from Pigs and Humans. Microbiology.

[46] Aguirre L., Vidal A., Seminati C., Tello M., Redondo N., Darwich L., Martín M. (2020). Antimicrobial Resistance Profile and Prevalence of Extended-Spectrum Beta-Lactamases (ESBL), AmpC Beta-Lactamases and Colistin Resistance (*mcr*) Genes in *Escherichia coli* from Swine between 1999 and 2018. Porc. Health Manag..

[47] Trongjit S., Chuanchuen R. (2021). Whole Genome Sequencing and Characteristics of *Escherichia coli* with Co-Existence of ESBL and Mcr Genes from Pigs. PLoS ONE.

[48] Alcock B.P., Raphenya A.R., Lau T.T.Y., Tsang K.K., Bouchard M., Edalatmand A., Huynh W., Nguyen A.-L.V., Cheng A.A., Liu S. (2020). CARD 2020: Antibiotic Resistome Surveillance with the Comprehensive Antibiotic Resistance Database. Nucleic Acids Res..

[49] Johansson M.H.K., Bortolaia V., Tansirichaiya S., Aarestrup F.M., Roberts A.P., Petersen T.N. (2021). Detection of Mobile Genetic Elements Associated with Antibiotic Resistance in Salmonella enterica Using a Newly Developed Web Tool: MobileElementFinder. J. Antimicrob. Chemother..

[50] Krawczyk P.S., Lipinski L., Dziembowski A. (2018). PlasFlow: Predicting Plasmid Sequences in Metagenomic Data Using Genome Signatures. Nucleic Acids Res..

[51] Wu S., Dalsgaard A., Hammerum A.M., Porsbo L.J., Jensen L.B. (2010). Prevalence and Characterization of Plasmids Carrying Sulfonamide Resistance Genes among *Escherichia coli* from Pigs, Pig Carcasses and Human. Acta Vet. Scand..

[52] Byrne-Bailey K.G., Gaze W.H., Kay P., Boxall A.B.A., Hawkey P.M., Wellington E.M.H. (2009). Prevalence of Sulfonamide Resistance Genes in Bacterial Isolates from Manured Agricultural Soils and Pig Slurry in the United Kingdom. Antimicrob. Agents Chemother..

[53] Bryan A., Shapir N., Sadowsky M.J. (2004). Frequency and Distribution of Tetracycline Resistance Genes in Genetically Diverse, Nonselected, and Nonclinical *Escherichia coli* Strains Isolated from Diverse Human and Animal Sources. Appl. Environ. Microbiol..

[54] Yue L., Jiang H.-X., Liao X.-P., Liu J.-H., Li S.-J., Chen X.-Y., Chen C.-X., Lü D.-H., Liu Y.-H. (2008). Prevalence of Plasmid-Mediated Quinolone Resistance qnr Genes in Poultry and Swine Clinical Isolates of *Escherichia coli*. Vet. Microbiol..

[55] Liu Y.-Y., Wang Y., Walsh T.R., Yi L.-X., Zhang R., Spencer J., Doi Y., Tian G., Dong B., Huang X. (2016). Emergence of Plasmid-Mediated Colistin Resistance Mechanism MCR-1 in Animals and Human Beings in China: A Microbiological and Molecular Biological Study. Lancet Infect. Dis..

[56] Aghapour Z., Gholizadeh P., Ganbarov K., Bialvaei A.Z., Mahmood S.S., Tanomand A., Yousefi M., Asgharzadeh M., Yousefi B., Kafil H.S. (2019). Molecular Mechanisms Related to Colistin Resistance in Enterobacteriaceae. Infect. Drug Resist..

[57] Feldgarden M., Brover V., Haft D.H., Prasad A.B., Slotta D.J., Tolstoy I., Tyson G.H., Zhao S., Hsu C.-H., McDermott P.F. (2019). Validating the AMRFinder Tool and Resistance Gene Database by Using Antimicrobial Resistance Genotype-Phenotype Correlations in a Collection of Isolates. Antimicrob. Agents Chemother..

[58] Kaspersen H.P., Brouwer M.S., Nunez-Garcia J., Cárdenas-Rey I., AbuOun M., Duggett N., Ellaby N., Delgado-Blas J., Hammerl J.A., Getino M. (2024). *Escherichia coli* from Six European Countries Reveals Differences in Profile and Distribution of Critical Antimicrobial Resistance Determinants within One Health Compartments, 2013 to 2020. Eurosurveillance.

[59] (2017). Methods for Antimicrobial Susceptibility Testing of Infrequently Isolated or Fastidious Bacteria Isolated from Animals.

[60] (2018). Methods for Dilution Antimicrobial Susceptibility Tests for Bacteria That Grow Aerobically.

[61] Shen Z. (2025). DNA Extraction with Zymo Quick-DNA^TM^ Fungal/Bacterial Miniprep Kit. https://www.protocols.io/view/dna-extraction-with-zymo-quick-dna-fungal-bacteria-dm6gpdw98gzp/v1.

[62] Zeden M.S., Gründling A. (2023). Small-Scale Illumina Library Preparation Using the Illumina Nextera XT DNA Library Preparation Kit. Cold Spring Harb. Protoc..

[63] Andrews S. FastQC A Quality Control Tool for High Throughput Sequence Data. https://www.bioinformatics.babraham.ac.uk/projects/fastqc/.

[64] Chen S., Zhou Y., Chen Y., Gu J. (2018). Fastp: An Ultra-Fast All-in-One FASTQ Preprocessor. Bioinformatics.

[65] Benoit G., Lavenier D., Lemaitre C., Rizk G. (2014). Bloocoo, a Memory Efficient Read Corrector.

[66] Krueger F. (2022). Trim Galore. https://www.bioinformatics.babraham.ac.uk/projects/trim_galore/.

[67] Li D., Liu C.-M., Luo R., Sadakane K., Lam T.-W. (2015). MEGAHIT: An Ultra-Fast Single-Node Solution for Large and Complex Metagenomics Assembly via Succinct *de Bruijn* Graph. Bioinformatics.

[68] Vasilinetc I., Prjibelski A.D., Gurevich A., Korobeynikov A., Pevzner P.A. (2015). Assembling Short Reads from Jumping Libraries with Large Insert Sizes. Bioinformatics.

[69] Gurevich A., Saveliev V., Vyahhi N., Tesler G. (2013). QUAST: Quality Assessment Tool for Genome Assemblies. Bioinformatics.

[70] Manni M., Berkeley M.R., Seppey M., Simão F.A., Zdobnov E.M. (2021). BUSCO Update: Novel and Streamlined Workflows along with Broader and Deeper Phylogenetic Coverage for Scoring of Eukaryotic, Prokaryotic, and Viral Genomes. Mol. Biol. Evol..

[71] Vurture G.W., Sedlazeck F.J., Nattestad M., Underwood C.J., Fang H., Gurtowski J., Schatz M.C. (2017). GenomeScope: Fast Reference-Free Genome Profiling from Short Reads. Bioinformatics.

[72] Parks D.H., Imelfort M., Skennerton C.T., Hugenholtz P., Tyson G.W. (2015). CheckM: Assessing the Quality of Microbial Genomes Recovered from Isolates, Single Cells, and Metagenomes. Genome Res..

[73] Wood D.E., Salzberg S.L. (2014). Kraken: Ultrafast Metagenomic Sequence Classification Using Exact Alignments. Genome Biol..

[74] Hyatt D., Chen G.-L., Locascio P.F., Land M.L., Larimer F.W., Hauser L.J. (2010). Prodigal: Prokaryotic Gene Recognition and Translation Initiation Site Identification. BMC Bioinform..

