# Supplementary material for: Inferred Mobility-Resolved Resistome Architecture Suggests Recurrent Co-Resistance Modules on a Conserved Chromosomal Backbone in Multidrug-Resistant Escherichia coli from Intensive Swine Production in Hungary
Source: Antibiotics (Basel). 2026 Apr 2;15(4):367. doi: 10.3390/antibiotics15040367 (PMC13113209; doi:10.3390/antibiotics15040367)
Supplement: Supplementary file 1 [file antibiotics-15-00367-s001.zip › antibiotics-4185849-supplementary.pdf]

**Supplementary Table S1** Genomic context of non-β-lactam resistance determinants by antimicrobial class in the whole genome sequencing (WGS) cohort. Counts indicate the number of detected resistance determinants assigned to the chromosome, mobile genetic element (MGE; within 10 kb of an element identified by MobileElementFinder), or plasmid (PlasFlow-predicted plasmid contigs, ≥10 kb criterion). Classes are shown for acquired/non-β-lactam resistance markers; totals reflect gene hits aggregated across genomes.

| **Class** | **Chromosome** | **MGE** | **Plasmid** |
|---|---|---|---|
| Tetracycline | 3 | 12 | 33 |
| Sulfonamide | 3 | 2 | 31 |
| Trimethoprim | 0 | 2 | 22 |
| Aminoglycosides | 7 | 10 | 60 |
| Phenicols | 1 | 2 | 17 |
| Fluoroquinolones (PMQR) | 0 | 0 | 2 |
| Polymyxins (mcr) | 0 | 0 | 3 |
| Macrolides | 118 | 0 | 6 |

**Supplementary Table S2** Key acquired antimicrobial resistance determinants and genomic context in the whole genome sequencing (WGS) cohort. The table summarizes major acquired resistance genes by antimicrobial class and their inferred genomic location. Isolates positive indicates the number of genomes carrying the given gene (≥1 occurrence), and Percent isolates is calculated relative to the total WGS cohort. Genomic context was assigned using a hierarchical rule: genes were classified as mobile genetic element (MGE)-associated if located within 10 kb of a mobile element detected by MobileElementFinder; otherwise as plasmid-associated if located on PlasFlow-predicted plasmid contigs (≥10 kb criteria); remaining hits were considered chromosomal. Chromosome/Plasmid/MGE report isolate-level counts per compartment, and Total hits denote the total number of occurrences across genomes (multiple hits per isolate may occur).

| Gene | Class | Isolates positive | Percent isolates | Chromosome | Plasmid | MGE | Total hits |
|---|---|---|---|---|---|---|---|
| *ANT(3'')-IIa* | Aminoglycosides | 25 | 20.8 | 4 | 16 | 5 | 25 |
| *APH(3'')-Ib* | Aminoglycosides | 18 | 15.0 | 0 | 16 | 2 | 18 |
| *APH(6)-Id* | Aminoglycosides | 16 | 13.3 | 0 | 15 | 1 | 16 |
| *ANT(2'')-Ia* | Aminoglycosides | 6 | 5.0 | 3 | 3 | 0 | 6 |
| *APH(3')-Ia* | Aminoglycosides | 6 | 5.0 | 0 | 6 | 0 | 6 |
| *aadA2* | Aminoglycosides | 3 | 2.5 | 0 | 2 | 1 | 3 |
| *AAC(3)-VIa* | Aminoglycosides | 2 | 1.7 | 0 | 1 | 1 | 2 |
| *AAC(3)-IIe* | Aminoglycosides | 1 | 0.8 | 0 | 1 | 0 | 1 |
| *qnrB5* | Fluoroquinolones (PMQR) | 2 | 1.7 | 0 | 2 | 0 | 2 |
| *mphB* | Macrolides | 118 | 98.3 | 116 | 2 | 0 | 120 |
| *mphA* | Macrolides | 4 | 3.3 | 0 | 4 | 0 | 4 |
| *catI* | Phenicols | 11 | 9.2 | 1 | 9 | 1 | 11 |
| *floR* | Phenicols | 9 | 7.5 | 0 | 8 | 1 | 9 |
| *mcr-1* | Polymyxins (*mcr*) | 3 | 2.5 | 0 | 3 | 0 | 3 |
| *sul1* | Sulfonamide | 17 | 14.2 | 0 | 16 | 1 | 17 |
| *sul2* | Sulfonamide | 13 | 10.8 | 0 | 12 | 1 | 13 |
| *sul3* | Sulfonamide | 6 | 5.0 | 3 | 3 | 0 | 6 |
| *tet(A)* | Tetracycline | 30 | 25.0 | 0 | 19 | 11 | 30 |
| *tet(B)* | Tetracycline | 12 | 10.0 | 2 | 9 | 1 | 12 |
| *tet(D)* | Tetracycline | 4 | 3.3 | 1 | 3 | 0 | 4 |
| *tet(C)* | Tetracycline | 2 | 1.7 | 0 | 2 | 0 | 2 |
| *dfrA1* | Trimethoprim | 17 | 14.2 | 0 | 17 | 0 | 17 |
| *dfrA5* | Trimethoprim | 6 | 5.0 | 0 | 4 | 2 | 6 |
| *dfrA14* | Trimethoprim | 1 | 0.8 | 0 | 1 | 0 | 1 |

**Supplementary Table S3** Mobile genetic element (MGE)-associated acquired antimicrobial resistance genes (ARGs). ARGs were classified as MGE-associated when located within 10 kb of a mobile element detected by MobileElementFinder. "Mobile element type" denotes the element category reported by MobileElementFinder. Hits represent the total number of MGE-proximal ARG occurrences across all genomes (multiple hits per genome are possible). Isolates indicate the number of genomes carrying at least one MGE-proximal occurrence of the given ARG. Plasmid hits denote the subset of hits located on contigs predicted as plasmid by PlasFlow (≥10 kb criterion).

| **Gene** | **Mobile element type** | **Hits** | **Isolates** | **Plasmid hits** |
|---|---|---|---|---|
| *tet(A)* | unit transposon | 11 | 11 | 11 |
| *ANT(3'')-IIa* | unit transposon | 4 | 4 | 1 |
| *APH(3'')-Ib* | unit transposon | 2 | 2 | 2 |
| *AAC(3)-VIa* | insertion sequence | 1 | 1 | 1 |
| *ANT(3'')-IIa* | insertion sequence | 1 | 1 | 1 |
| *APH(6)-Id* | unit transposon | 1 | 1 | 1 |
| *aadA2* | unit transposon | 1 | 1 | 1 |
| *catI* | insertion sequence | 1 | 1 | 1 |
| *dfrA5* | insertion sequence | 1 | 1 | 1 |
| *dfrA5* | unit transposon | 1 | 1 | 1 |
| *floR* | insertion sequence | 1 | 1 | 1 |
| *sul1* | insertion sequence | 1 | 1 | 1 |
| *sul2* | unit transposon | 1 | 1 | 1 |
| *tet(B)* | insertion sequence | 1 | 1 | 1 |

**Supplementary Table S4** Phenotype–genotype agreement metrics for whole-genome sequencing (WGS)-based resistance prediction in the WGS-linked subset. Phenotypic non-susceptibility was defined from broth microdilution MIC data using the interpretive criteria applied in this study. Genotypic predictions were based on presence/absence of acquired resistance determinants detected with CARD-based workflows. For folate-pathway inhibitors, prediction required concurrent detection of *sul* (*sul1*/*sul2*/*sul3*) and *dfrA* genes. Performance metrics are reported as sensitivity (TP/[TP+FN]), specificity (TN/[TN+FP]) and accuracy ([TP+TN]/N). Discordances may reflect chromosomal mechanisms (e.g., target mutations, efflux/regulatory changes), gene expression context, or breakpoint-adjacent MIC distributions.

| Antimicrobial class | Genotypic prediction rule | N | Sensitivity (%) | Specificity (%) | PPV (%) | NPV (%) | Accuracy (%) | Notes |
|---|---|---|---|---|---|---|---|---|
| Tetracyclines | Any *tet* gene detected (e.g., *tetA*/*B*/*C*/*D*) | 116 | 69.6 | 91.7 | 88.6 | 76.4 | 81.0 | Class-dependent agreement; chromosomal regulators/efflux may contribute to discordance. |
| Folate-pathway inhibitors (TMP–SMX / potentiated sulfonamide) | Concurrent detection of ≥1 *sul* (*sul1*/*2*/*3*) AND ≥1 *dfrA* gene | 116 | 43.3 | 97.7 | 96.7 | 83.2 | 83.6 | Requiring *sul*+*dfrA* increases specificity; reflects co-selection modules. |
| Aminoglycosides | Any aminoglycoside-modifying enzyme gene (*aac*/*aph*/*ant*/*aad*) | 116 | 45.8 | 78.3 | 35.5 | 84.7 | 71.6 | Weaker agreement is likely due to heterogeneous gene content and context effects. |
| Polymyxins (colistin) | *mcr-1* detected | 116 | 42.9 | 100.0 | 100.0 | 96.5 | 96.6 | *mcr-1* explains a minority of phenotypic non-susceptibility; remaining cases are likely chromosomal. |

**Supplementary Table S5.** Farm- and age-group associations for multidrug-resistant (MDR) and selected phenotypic endpoints. Associations were tested using $\chi^2$ tests (two-sided). P-values were adjusted using the Benjamini–Hochberg procedure (BH-FDR). Endpoint definitions are provided in the table. DOX: doxycycline, GEN: gentamicin, NEO: neomycin, TMP: trimethoprim, SMX: sulfamethoxazole, FLO: florfenicol, ENR: enrofloxacin, MAR: marbofloxacin, COL: colistin

**Panel A: Farm associations**

| Endpoint | Definition | Farm 1 n/N (%) | Farm 2 n/N (%) | Farm 3 n/N (%) | Farm 4 n/N (%) | p | q (BH-FDR) | CramersV |
|---|---|---|---|---|---|---|---|---|
| **MDR (≥3 classes)** | Non-susceptible in ≥3 non-β-lactam classes (see Methods) | 39/58 (67.2) | 16/70 (22.9) | 9/39 (23.1) | 14/36 (38.9) | 6.96e-07 | 1.19e-06 | 0.393 |
| **Tetracyclines (DOX)** | Non-susceptible if DOX ≥16 μg/mL | 41/58 (70.7) | 30/70 (42.9) | 10/39 (25.6) | 14/36 (38.9) | 7.71e-05 | 0.000103 | 0.327 |
| **Aminoglycosides (GEN/NEO)** | Non-susceptible if GEN ≥16 μg/mL or NEO ≥32 μg/mL | 30/58 (51.7) | 4/70 (5.7) | 1/39 (2.6) | 19/36 (52.8) | 1.19e-12 | 4.76e-12 | 0.537 |
| **Folate inhibitors (TMP–SMX)** | Non-susceptible if PSA ≥4 μg/mL | 31/58 (53.4) | 13/70 (18.6) | 10/39 (25.6) | 12/36 (33.3) | 0.000325 | 0.00039 | 0.303 |
| **Phenicols (FLO)** | Non-susceptible if FLO ≥8 μg/mL | 40/58 (69.0) | 39/70 (55.7) | 28/39 (71.8) | 30/36 (83.3) | 0.0302 | 0.0302 | 0.210 |
| **Fluoroquinolones (ENR/MAR)** | Non-susceptible if ENR ≥0.25 μg/mL or MAR ≥0.25 μg/mL | 37/58 (63.8) | 13/70 (18.6) | 8/39 (20.5) | 5/36 (13.9) | 6.16e-09 | 1.85e-08 | 0.450 |
| **Polymyxins (colistin)** | Non-susceptible if COL ≥8 μg/mL | 31/58 (53.4) | 3/70 (4.3) | 2/39 (5.1) | 0/36 (0.0) | 2.15e-15 | 2.57e-14 | 0.593 |
| **Colistin high-MIC tail** | High tail defined as COL ≥16 μg/mL | 29/58 (50.0) | 3/70 (4.3) | 1/39 (2.6) | 0/36 (0.0) | 1.02e-14 | 6.1e-14 | 0.580 |
| **Enrofloxacin high-MIC tail** | High tail defined as ENR ≥32 μg/mL | 14/58 (24.1) | 0/70 (0.0) | 0/39 (0.0) | 0/36 (0.0) | 3.45e-08 | 8.27e-08 | 0.430 |
| **Marbofloxacin high-MIC tail** | High tail defined as MAR ≥32 μg/mL | 12/58 (20.7) | 0/70 (0.0) | 0/39 (0.0) | 0/36 (0.0) | 5.53e-07 | 1.11e-06 | 0.396 |
| **TMP–SMX high-MIC tail** | High tail defined as PSA ≥256 μg/mL | 26/58 (44.8) | 13/70 (18.6) | 8/39 (20.5) | 7/36 (19.4) | 0.0031 | 0.00338 | 0.261 |
| **Doxycycline high-MIC tail** | High tail defined as DOX ≥128 μg/mL | 17/58 (29.3) | 3/70 (4.3) | 1/39 (2.6) | 8/36 (22.2) | 4.81e-05 | 7.21e-05 | 0.334 |

**Panel B: Age-group associations**

| Endpoint | Definition | one-day n/N (%) | 4 weeks n/N (%) | 6 weeks n/N (%) | p | q (BH-FDR) | CramersV |
|---|---|---|---|---|---|---|---|
| **MDR (≥3 classes)** | Non-susceptible in ≥3 non-β-lactam classes (see Methods) | 30/72 (41.7) | 23/65 (35.4) | 25/66 (37.9) | 0.747 | 0.837 | 0.054 |
| **Tetracyclines (DOX)** | Non-susceptible if DOX ≥16 μg/mL | 39/72 (54.2) | 26/65 (40.0) | 30/66 (45.5) | 0.244 | 0.365 | 0.118 |
| **Aminoglycosides (GEN/NEO)** | Non-susceptible if GEN ≥16 μg/mL or NEO ≥32 μg/mL | 17/72 (23.6) | 18/65 (27.7) | 19/66 (28.8) | 0.767 | 0.837 | 0.051 |
| **Folate inhibitors (TMP–SMX)** | Non-susceptible if PSA ≥4 μg/mL | 26/72 (36.1) | 19/65 (29.2) | 21/66 (31.8) | 0.684 | 0.837 | 0.061 |

| | | | | | | | |
|---|---|---|---|---|---|---|---|
| **Phenicols (FLO)** | Non-susceptible if FLO ≥8 μg/mL | 63/72 (87.5) | 40/65 (61.5) | 34/66 (51.5) | 1.79e-05 | 0.000214 | 0.328 |
| **Fluoroquinolones (ENR/MAR)** | Non-susceptible if ENR ≥0.25 μg/mL or MAR ≥0.25 μg/mL | 16/72 (22.2) | 25/65 (38.5) | 22/66 (33.3) | 0.108 | 0.357 | 0.148 |
| **Polymyxins (colistin)** | Non-susceptible if COL ≥8 μg/mL | 9/72 (12.5) | 16/65 (24.6) | 11/66 (16.7) | 0.173 | 0.357 | 0.132 |
| **Colistin high-MIC tail** | High tail defined as COL ≥16 μg/mL | 8/72 (11.1) | 16/65 (24.6) | 9/66 (13.6) | 0.0793 | 0.357 | 0.158 |
| **Enrofloxacin high-MIC tail** | High tail defined as ENR ≥32 μg/mL | 5/72 (6.9) | 7/65 (10.8) | 2/66 (3.0) | 0.217 | 0.365 | 0.123 |
| **Marbofloxacin high-MIC tail** | High tail defined as MAR ≥32 μg/mL | 5/72 (6.9) | 6/65 (9.2) | 1/66 (1.5) | 0.156 | 0.357 | 0.135 |
| **TMP–SMX high-MIC tail** | High tail defined as PSA ≥256 μg/mL | 19/72 (26.4) | 17/65 (26.2) | 18/66 (27.3) | 0.988 | 0.988 | 0.011 |
| **Doxycycline high-MIC tail** | High tail defined as DOX ≥128 μg/mL | 13/72 (18.1) | 5/65 (7.7) | 11/66 (16.7) | 0.178 | 0.357 | 0.130 |

**Supplementary Table S6.** Effect-size analyses for farm- and age-associated phenotypic endpoints (multivariable logistic regression) and farm-level prevalence/associations of key acquired antimicrobial resistance gene (ARG) groups in the whole-genome sequencing (WGS) subset. MDR: multidrug resistant. MIC: minimum inhibitory concentration. TMP: trimethoprim. SMX: sulfamethoxazole.

**Panel A: Adjusted farm/age effects**

| Outcome | Predictor | OR | 95% CI | p |
|---|---|---|---|---|
| **MDR (≥3 non-susceptible classes)** | Farm 1 - Farm 2 | 0.145826195 | 0.0666–0.319 | 1.46263E-06 |
| **MDR (≥3 non-susceptible classes)** | Farm 1 - Farm 3 | 0.146101709 | 0.0579–0.369 | 4.64836E-05 |
| **MDR (≥3 non-susceptible classes)** | Farm 1 - Farm 4 | 0.312635508 | 0.131–0.744 | 0.008575048 |
| **MDR (≥3 non-susceptible classes)** | Age 4 weeks | 0.818111121 | 0.385–1.74 | 0.601062511 |
| **MDR (≥3 non-susceptible classes)** | Age 6 weeks | 0.90749837 | 0.431–1.91 | 0.798317961 |
| **Colistin high-MIC tail (MIC ≥16 μg/mL)** | Age 4 weeks | 7.009137356 | 1.96–25.1 | 0.002767666 |
| **Colistin high-MIC tail (MIC ≥16 μg/mL)** | Age 6 weeks | 1.732892887 | 0.521–5.76 | 0.369736681 |
| **Colistin high-MIC tail (MIC ≥16 μg/mL)** | Farm 1 vs others | 54.78314511 | 15.6–193 | 4.62326E-10 |
| **TMP–SMX high-MIC tail (MIC ≥256 μg/mL)** | Farm 1 - Farm 2 | 0.278450124 | 0.126–0.618 | 0.001662248 |
| **TMP–SMX high-MIC tail (MIC ≥256 μg/mL)** | Farm 1 - Farm 3 | 0.317190859 | 0.125–0.807 | 0.01598402 |
| **TMP–SMX high-MIC tail (MIC ≥256 μg/mL)** | Farm 1 - Farm 4 | 0.295236522 | 0.111–0.783 | 0.014258134 |
| **TMP–SMX high-MIC tail (MIC ≥256 μg/mL)** | Age 4 weeks | 1.075429055 | 0.487–2.37 | 0.857132714 |
| **TMP–SMX high-MIC tail (MIC ≥256 μg/mL)** | Age 6 weeks | 1.125360424 | 0.514–2.46 | 0.767604067 |
| **Doxycycline high-MIC tail (MIC ≥128 μg/mL)** | Farm 1 - Farm 2 | 0.108687612 | 0.0297–0.397 | 0.000792059 |
| **Doxycycline high-MIC tail (MIC ≥128 μg/mL)** | Farm 1 - Farm 3 | 0.061851912 | 0.0078–0.491 | 0.00843906 |
| **Doxycycline high-MIC tail (MIC ≥128 μg/mL)** | Farm 1 - Farm 4 | 0.706908462 | 0.264–1.89 | 0.489419743 |
| **Doxycycline high-MIC tail (MIC ≥128 μg/mL)** | Age 4 weeks | 0.37577551 | 0.12–1.18 | 0.093395435 |
| **Doxycycline high-MIC tail (MIC ≥128 μg/mL)** | Age 6 weeks | 0.966823522 | 0.374–2.5 | 0.944487661 |

**Panel B: ARG groups by farm**

| Gene group | p ($\chi^2$) | Cramér's V | Farm 1 | Farm 2 | Farm 3 | Farm 4 |
|---|---|---|---|---|---|---|
| **Tetracyclines (*tet*)** | 0.039349857 | 0.268256169 | 16/26 (61.5%) | 16/52 (30.8%) | 4/10 (40.0%) | 8/28 (28.6%) |
| **Sulfonamides (*sul*)** | 0.145144974 | 0.215629908 | 9/26 (34.6%) | 7/52 (13.5%) | 3/10 (30.0%) | 5/28 (17.9%) |
| **Trimethoprim (*dfrA*)** | 0.938521253 | 0.059328613 | 4/26 (15.4%) | 11/52 (21.2%) | 2/10 (20.0%) | 5/28 (17.9%) |
| **Aminoglycosides (AMEs)** | 0.003443154 | 0.342871913 | 14/26 (53.8%) | 8/52 (15.4%) | 3/10 (30.0%) | 6/28 (21.4%) |
| **Phenicols (*floR*/*catI*)** | 0.348755014 | 0.168462082 | 3/26 (11.5%) | 4/52 (7.7%) | 0/10 (0.0%) | 5/28 (17.9%) |
| **PMQR (*qnrB5*)** | 0.474438074 | 0.146943672 | 0/26 (0.0%) | 2/52 (3.8%) | 0/10 (0.0%) | 0/28 (0.0%) |
| **Polymyxins (*mcr-1*)** | 0.013711918 | 0.303148893 | 3/26 (11.5%) | 0/52 (0.0%) | 0/10 (0.0%) | 0/28 (0.0%) |
